# SARS-CoV-2-Induced Myocarditis: A State-of-the-Art Review

**DOI:** 10.3390/v15040916

**Published:** 2023-04-02

**Authors:** Francesco Nappi, Sanjeet Singh Avtaar Singh

**Affiliations:** 1 Department of Cardiac Surgery, Centre Cardiologique du Nord, 93200 Saint-Denis, France; 2 Department of Cardiothoracic Surgery, Royal Infirmary of Edinburgh, Edinburgh EH16 4SA, UK

**Keywords:** myocarditis, COVID-19, SARS-CoV-2-induced myocarditis, endomyocardial biopsy, autopsy

## Abstract

In this review, we investigated whether severe acute respiratory syndrome coronavirus 2 (SARS-CoV-2) can directly cause myocarditis with severe myocardial damage induced by viral particles. A review of the major data published from 2020 to 2022 was performed by consulting the major databases alongside first-hand experiences that emerged from the cardiac biopsies and autopsy examinations of patients who died of SARS-CoV-2 infections. From this study, a significantly large amount of data suggests that the Dallas criteria were met in a residual percentage of patients, demonstrating that SARS-CoV-2 myocarditis was a rare clinical and pathological entity that occurred in a small percentage of subjects. All cases described here were highly selected and subjected to autopsies or endomyocardial biopsies (EMBs). The most important discovery, through the detection of the SARS-CoV-2 genome using the polymerase chain reaction, consisted in the presence of the viral genome in the lung tissue of most of the patients who died from COVID-19. However, the discovery of the SARS-CoV-2 viral genome was a rare event in cardiac tissue from autopsy findings of patients who died of myocarditis It is important to emphasize that myocardial inflammation alone, as promoted by macrophages and T cell infiltrations, can be observed in noninfectious deaths and COVID-19 cases, but the extent of each cause is varied, and in neither case have such findings been reported to support clinically relevant myocarditis. Therefore, in the different infected vs. non-infected samples examined, none of our findings provide a definitive histochemical assessment for the diagnosis of myocarditis in the majority of cases evaluated. We report evidence suggesting an extremely low frequency of viral myocarditis that has also been associated with unclear therapeutic implications. These two key factors strongly point towards the use of an endomyocardial biopsy to irrefutably reach a diagnosis of viral myocarditis in the context of COVID-19.

## 1. Definition

**Figure 1:**
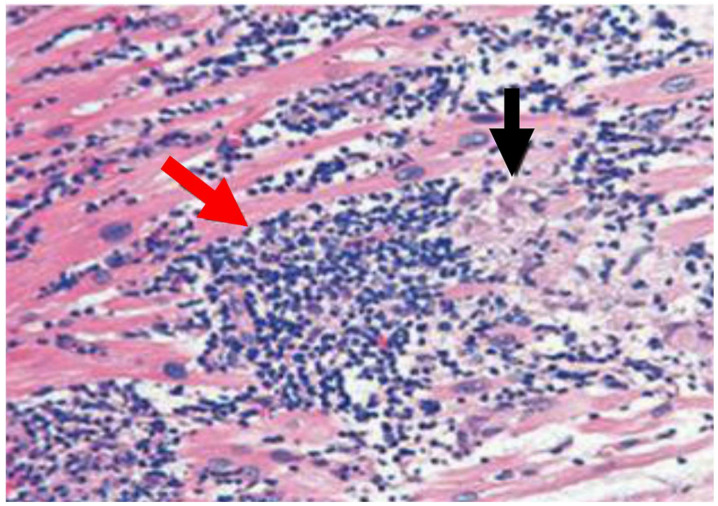
Image of patients with active myocarditis that is suggestive of a lymphocytic and histiocytic infiltrate and higher T lymphocytes in heart-tissue sections. Hematoxylin and eosin immunohistochemistry of heart tissue samples depicts characteristic lesions of acute myocarditis with widespread lymphocytic and histiocytic infiltrate (red arrow) and associated myocyte damage (black arrow).

The definition of myocarditis is supported by the Dallas Standard Pathologic Criteria, which states that it is mandatory to demonstrate the existence of an inflammatory cellular infiltrate with or without associated myocyte necrosis. Evaluation of the infiltrate is performed on conventionally stained sections of cardiac tissue (Figure 1) [1,2]. 

**Figure 2:**
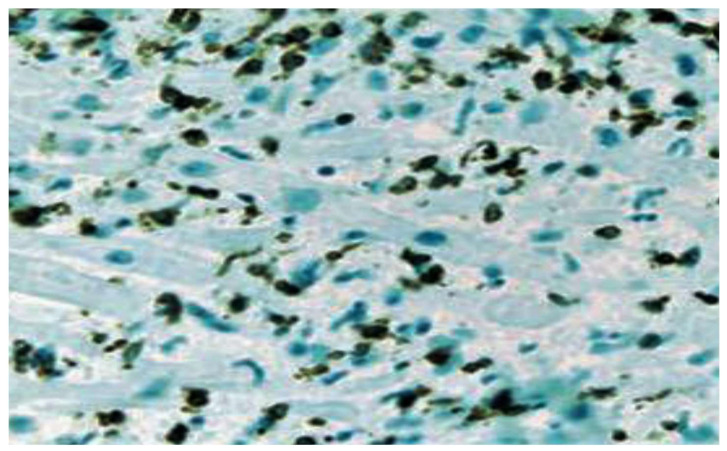
Borderline myocarditis characterized by the infiltration of lymphocytic and histiocytic cells is depicted using CD3 immunostaining of T lymphocytes.

However, these criteria may be constrained by variability in interpretation, inadequacy of prognostic effectiveness, and poor sensitivity. Sampling errors cannot be excluded [3,4,5], and Baughman and colleagues (2006) emphasized “the death of the Dallas criteria” [6]. Concerns related to these limitations have resulted in alternative pathological classifications that rely on criteria dictated by cell-specific immunoperoxidase stains directed to surface antigens, such as anti-CD3, anti-CD4, anti-CD20, anti-CD68, and anti-human leukocyte antigen (Figure 2) [7,8,9,10]. 

As such, in terms of precision, the criteria based on immunoperoxidase staining have higher sensitivity, conferring prognostic value [11,12]. Several studies have suggested the substantial role of non-invasive cardiac magnetic resonance imaging (CMRI), which is the reference standard for diagnosis, bypassing the risks associated with cardiac biopsies. With the use of cardiac MRIs, regions of myocarditis have been observed to closely correlate with regions of abnormal signal [13,14,15,16]. The current recommendations are proposed on the basis of consensus emerging from the position statement that invasive studies such as endomyocardial biopsies (EMBs) should be performed only if there is a good overall prognosis for patients with mild acute dilated cardiomyopathy, even if these subjects are potentially at risk of worsening clinical conditions. In this patient population, an endomyocardial biopsy should be reserved if there is a likelihood of finding specific treatable disorders [17,18,19].

During the SARS-CoV-2 pandemic, patients with COVID-19 and predictors of a poor prognosis were referred to doctors who sought evidence to arrive at a diagnosis and initiate treatment according to the criteria described above, often experiencing difficulty [17]. The balance sheet after the pandemic has suggested that clinicians saw a considerable number of patients with non-specific cardiovascular symptoms associated with non-specific CMR findings.

## 2. Methods

This narrative review was carried out from December 2022 to January 2023. PubMed, MEDLINE, Embase, and the Cochrane Library were searched using the following terms: “myocarditis” or “viral myocarditis”, “SARS-CoV-2 myocarditis”, “COVID-19 myocarditis” together with “SARS-CoV-2 epidemiology”, “COVID-19 epidemiology”, “SARS-CoV-2 pathogenesis”, “COVID-19 pathogenesis”, “SARS-CoV-2 manifestations”, and “COVID-19 manifestations.” The reference list of articles identified by this search strategy was noted, and articles that were considered relevant were screened. Publications selected were primarily from the past two years. Highly regarded manuscripts and widely referenced articles outside this timeline were not excluded. Some recommended review articles were mentioned to provide readers with additional information and background references. Only articles in the English language were included.

## 3. The Clinical Problem

Infection caused by SARS-CoV-2 may cause considerable morbidity and mortality. Former epidemiological evidence suggests that roughly 20% of patients who require hospitalization have proof of cardiac damage, as indicated by increased levels of high-sensitivity troponins (hs-cTnI) [20]. Lala et al. reported [21] that plasma levels of troponin reached 36% within 24 h of hospitalization to estimate cardiac injury. Multivariate logistic regression for disease severity and relevant risk factor dissimilarities noted that even small areas of damaged myocardium were linked with higher mortality. Nonetheless, considerable key unresolved issues persist about the characteristics of myocardial damage in patients with COVID-19. The veil of uncertainty focuses on the absence of obstructive epicardial coronary artery disease, which is supported by the expression of myocardial damage, explained by the presence of positive troponins with or without wall motion abnormalities [17]. In this context, clinicians are often prompted to diagnose acute myocarditis by default, interpreting it as an underlying cause and using clinical and imaging markers of myocyte damage as data. In fact, several reports have shown that most cases labeled as myocarditis by COVID-19 infections were due to abnormal troponin plasma levels or cardiac magnetic resonance imaging (CMR) without immunopathological confirmation [22,23,24]. It is noted with concern that there is an absence of evidence to address this type of myocarditis (in the context of SARS-CoV-2), which persists, especially in the ‘restricted’ group [25,26]. Thus, a comprehensive interpretation of the pathogenesis of cardiac injury linked to COVID-19 infections is crucial for the development of suitable therapies. 

## 4. Pathophysiology and Effects of COVID-19-Induced Myocyte Infection/Injury

Acute myocarditis is a disease with a swinging clinical development and representation, which isolates it as an outlier as one of the most demanding diseases from a cardiology perspective. Several mechanisms have been hypothesized that are implicated in the pathogenesis of myocarditis and favored by COVID-19. Putting it into context, there is extremely little proof to sustain sudden cardiomyocyte destruction directly exerted by virus-mediated lysis with damage to cardiac frames, resulting in myocyte injury and cardiac disorder. Lindner and colleagues [25] examined 39 autopsy results from deceased patients in Germany to quantify viral load in cardiac autopsy sections and reported the presence of the virus in 24 of 39 (61.5%) recovered hearts, albeit without being able to affirm the cardiotropic nature of the virus (Table 1). From 16 out of 39 samples (41%), the copy number was greater than 1000 copies per ug of RNA [25]. Data from the viral replication analysis suggested that SARS-CoV-2, defined by the detection of the replicated (−) strand of the RNA genome, was demonstrated in 5 of the patients in whom the highest viral load occurred. However, on subsequent in situ hybridization, the presence of the virus was noted in the interstitial cells within the cardiac tissue but was not confirmed in the myocytes. The most disconcerting finding was that the presence of the virus was not substantially related to a greater infiltration of mononuclear cells in the myocardium. Furthermore, none of the biopsy findings of SARS-CoV-2-induced myocarditis were the pathoanatomical lesions described in the Dallas criteria. Another alternative mechanism potentially capable of promoting cardiac damage, which has been persistently proposed, directs the pathogenesis towards the direct entry of the virus into endothelial cells in the heart without a necessary explicit interaction between SARS-CoV-2 and myocardiocytes. In fact, several studies have reported that endothelial infection can be directly observed in cardiac autopsy sections as well as in those of glomerular endothelial cells by means of electron microscopy along with the distinct identification of viral particles. However, in some cases, their aspect and position within cells were not representative of coronavirus-infected cells [27,28]. Varga and colleagues [27], in a landmark publication in the Lancet in early 2020, evaluated the pathological alteration of the endothelium struck by a SARS-CoV-2 infection in a patient undergoing a renal transplant associated with coronary artery disease and arterial hypertension as important comorbidities. Electron microscopy of the specimens retrieved from the autopsy highlighted the presence of viral genomes in the endothelial cells of the transplanted kidney. The immunohistochemistry revealed an abundant accumulation of infiltrated inflammatory cells that were implicated in the endothelium of many tissues. Apoptotic bodies were recognized in the heart, small bowel, and lung. The lung revealed a considerable accumulation of mononuclear cells with a marked congestion involving small lung vessels. The authors deepened their examination of the cardiocirculatory systems of patients who died from irreversible forms of COVID-19. Of the three patients reported by Varga and colleagues, one showed severe right-sided heart failure with progression to left-sided ventricular failure preceded by ST-segment elevation and myocardial infarction. The immunohistochemical examination of the autopsy tissues was suggestive of the presence of severe lymphocytic endotheliitis in the lung, heart, kidney, and liver. The pathoanatomical cardiac damage revealed a myocardial infarction, albeit without any evidence of pathoanatomical lesions related to lymphocytic myocarditis. Liver cell necrosis resulted in a rapid evolution towards liver failure. The small intestine was analyzed, and severe endotheliitis of the submucosal vessels was observed [27].

This evidence supported the conclusion that other techniques, such as in situ hybridization, should be used to confirm the reported findings. Interestingly, Kawakami et al., using both techniques, were unable to document a single case of endothelial infection from SARS-CoV-2 in the heart [29]. Therefore, in consideration of all the data reported, it seems difficult to establish a definitive conclusion that identifies a decisive mechanism of COVID-19 capable of causing cardiac damage in endothelial tropism without adding other solid corroborating evidence. 

Another viable pathogenetic pathway, according to the literature, implicates the hyperactivation of the immune system as the trigger for heart damage, inducing the release of multiple inflammatory mediators, including interleukins and tumor necrosis factors [30,31,32,33,34,35,36]. The elevated circulating levels of these inflammatory mediators, which exceed normal thresholds, can cause collateral damage. The immune-inflammatory scenario dominated by these factors has been referred to as a “cytokine storm”, embodying the out-of-control inflammatory response that has been accurately described in patients with severe forms of COVID-19 [32,33,34,35,36]. Likewise, coagulation disorders resulting from the activation of platelets, neutrophils, and other proteins promote microvascular and macrovascular thrombi, contributing to vascular occlusion and cell death [37,38,39,40]. Guagliumi et al. [41] described a young female patient having COVID-19 with myocardial infarction and cardiogenic shock, caused by cardiac microvascular thrombi formation, who received urgent coronary angiography and primary percutaneous coronary intervention. The virus was yet to be highlighted by a polymerase chain reaction (PCR) test on cardiac findings, raising questions on the pathogenesis of cardiac damage induced by SARS-CoV-2. The authors speculated that many pathogenic dynamics of the inflammation/thrombosis interconnection remain to be elucidated during the COVID-19 infection. We observed a similar case in a 47-year-old woman without comorbid cardiovascular disease who, 10 days after a medium-severity form of COVID-19, was admitted to the hospital with a thrombotic occlusion of the right common carotid artery. Additionally, in this case, the patient had a negative result on a PCR test and was probably affected by the robust inflammatory response supported by high levels of cytokine production [42].

## 5. Clinical Evidence. The Concern of Diagnosis Related to COVID-19-Induced Myocardial Injury

The mechanisms underlying the organ damage caused by SARS-CoV-2 infections were not fully understood in the initial stages of the pandemic. Even today, more detailed answers on the best diagnostic approaches useful for identifying the causes responsible for the lesions that show heart disease in hospitalized and non-hospitalized patients are awaited. These responses suggested that in most cases, cardiac damage should be considered in the context of the overall respiratory infection rather than as an explanation of the prevailing display of the disease. Robust coagulation abnormalities established within the immune response to viral infection have proved that the venous and arterial circulation of COVID-19 patients were predisposed to thrombotic processes [43,44,45,46,47,48,49]. Choudhry and colleagues [48] evaluated patients who experienced COVID-19 and were hospitalized for ST-segment elevation myocardial infarction (STEMI) or patients with COVID-19 without STEMI. The authors reported that subjects with COVID-19 associated with STEMI had abnormally high troponin levels, the development of multivessel thrombi, and stent thrombosis compared to those COVID-19 patients in whom STEMI was not diagnosed. The same etiopathogenetic profile has been described in a patient with COVID-19 in whom multiple spontaneous coronary thromboses causing ST-elevation myocardial infarction were observed. A meta-analysis, based on real-world studies and adjusted estimates of risk, reported a survival benefit of therapeutic anticoagulation over prophylactic anticoagulation in COVID-19 patients [49].

However, a surprising finding that emerged was the absence of obvious coronary artery occlusion in a substantial number of the STEMI subjects studied. Bangalore and colleagues [50] collected their results from 18 hospitalized patients with a confirmed diagnosis of COVID-19 and associated ST-segment elevation. Investigators confirmed a diagnosis of acute coronary thrombosis causing myocardial infarction in only 44% of cases, while the other 56% of subjects revealed no coronary myocardial harm and were diagnosed with non-obstructive disease on coronary angiographies. Patients with an etiopathogenetic and symptomatic profile who report high plasma troponin levels but in whom the clinical suspicion of acute coronary artery disease is not confirmed represent the category of subjects in which diagnostic dilemmas can emerge. In fact, the clinical presentation of myocarditis is subtle, characterized by substantial variability, and may include indistinct values or non-specific symptoms. In these cases, patients complain of fatigue, dyspnea, palpitations, and chest discomfort, making the diagnosis of myocarditis difficult and confusing for those who experience coronary syndromes without persistent ST-segment elevation [20].

According to the World Health Organization, the definition of myocarditis includes an inflammatory disease of the myocardium for which the diagnosis is established with immunological and histological examinations supported by immunohistochemical and molecular criteria. The use of an endomyocardial biopsy (EMB) serves to gain certainty about the diagnosis and identify the potential cause of the disease. The recommendation to use an EMB is well established in the current European Society of Cardiology (ESC)/American Heart Association (AHA)/American College of Cardiology (ACC) guidelines and the position papers of professional societies based on clinical experience [10,17,19,51,52,53,54]. The ESC’s guideline recommendations for EMB [17] state that its use is motivated by “a strong reason to believe that the findings will have a significant effect on subsequent treatment decisions,” [10]. ESC guidelines in their Class 1a recommendation state that EMB should be performed in patients who have experienced the recent onset of heart failure symptoms associated with hemodynamic compromise or the occurrence of new ventricular arrhythmia episodes [17,19,54].

Although the use of IHC raises the sensitivity and specificity for myocarditis diagnosis [1,6,7], precise references are lacking to establish the exact timing and criteria for performing an EMB in cases of suspected myocarditis, especially after the recent impact that COVID-19 infections have had on cardiac damage.

In cases where necrosis is absent and the presence of lymphocytic infiltrates is not clearly documentable, the diagnosis of myocarditis takes on a borderline value. We also refer to the potential errors of sampling in the EMB containing viral pathogens detectable through the PCR method using the Dallas criteria, which brought to light negative results in 50% of the cases despite observing a viral infection [53]. Due to poor diagnostic evidence, the use of the Dallas criteria has been discouraged for the diagnosis of myocarditis.

Newly developed, robust immunohistochemical criteria have been evoked to address these shortcomings. Thus, immunohistochemistry strengthened the role of abnormal inflammatory lymphocytic infiltrates supporting myocarditis. The criteria establish that ≥14 leukocytes/mm^2^, including up to 4 monocytes/mm^2^, with the presence of CD3-positive T lymphocytes ≥ 7 cells/mm is diagnostic [54]. Evidence for the presence of myocytic degeneration and necrosis of non-ischemic origin must corroborate the cell counts in COVID-19-related myocarditis.

Anderson et al. [55] and Zhang and colleagues [56] noted that the appearance of inflammatory infiltrates alone with the absence of myocytic necrosis can be found in the myocardium of subjects who died of non-infectious causes. Ozieranski and colleagues [52,53] insisted on the inflammatory infiltrate model, whereby the extent of inflammatory damage and the presence of myocytic necrosis were needed to define a pathoanatomical picture of myocarditis. Again, the infiltrate must have the characteristics of a concentrated collection of inflammatory cells with a predominance of lymphocytes compared to macrophages that surround myocytes [52,53]. Histological evidence of myocarditis in association with viral PCR positivity allows us to reach a molecular biology-based diagnosis to confirm infective myocarditis. Therefore, a histologic picture of viral PCR-negative myocarditis defines myocarditis of non-viral etiology.

In clinical practice, EMB is not routinely used to arrive at the diagnosis of myocarditis and must be supported by the contributions of other noninvasive diagnostic tests in patients with suspected myocarditis due to COVID-19. Circumstantial evidence is provided by abnormal echocardiographic findings or MRIs of myocardial damage. Huang and colleagues [22] reported a series of 15 patients in whom cardiac involvement from SARS-CoV-2 was examined using cardiac magnetic resonance (CMR). Fifty-eight percent of patients studied who had cardiac symptoms but were considered cured of COVID-19 revealed substantial abnormalities on CMR imaging, mainly related to the presence of myocardial edema, fibrosis, and impaired right ventricular function. In another series, Puntmann and colleagues [23] evaluated a greater number of recovered patients (*n* = 100), recording cardiac involvement in 78% of them in CMR observations. About 75% of these subjects had markedly increased troponin levels [23]. These studies reflect the possibility that infections persist, even months after being considered recovered, with a previous unhealed myocardial inflammation that can evolve to left-sided ventricular dysfunction [52,53]. It is important to focus on data reporting on EMB outcomes in patients who experienced suspected COVID-19-induced myocarditis or those who disclosed unexplained heart failure. Furthermore, the poor overall performance offered by diagnostic investigations [57,58] and the role of EMB in cases of suspected COVID-19 myocarditis need to be discussed [1,17,18,19,20,52,53].

## 6. Pathogenesis of SARS-CoV-2-Induced Myocarditis

### 6.1. The SARS-CoV-2 Virus

Coronaviruses are a family of positive-sense single-stranded RNA viruses characterized by high heterogeneity. Our knowledge in the field of virology reports three widely documented human coronaviruses, which are highly pathogenic and have a lethal profile. These include SARS-CoV, Middle East respiratory syndrome-CoV, and SARS-CoV-2. There are differences in the genomic homology between these viruses. It has been observed that approximately 79.5% of the genetic makeup of SARS-CoV-2 is shared through genomic homology with SARS-CoV. This percentage is reduced to approximately 50% in the Middle East respiratory syndrome-CoV. For these genomic characteristics, the conclusion was reached that SARS-CoV-2 is genetically more similar to SARS-CoV [59]. Many likenesses between SARS-CoV and SARS-CoV-2 have been described, although crucial differences have affected the transmissibility and pathogenesis of the disease. The analysis of the effects of SARS-CoV has had some degree of relevance to our current understanding of how SARS-CoV-2 promotes its action on the cardiac structure.

**Figure 3:**
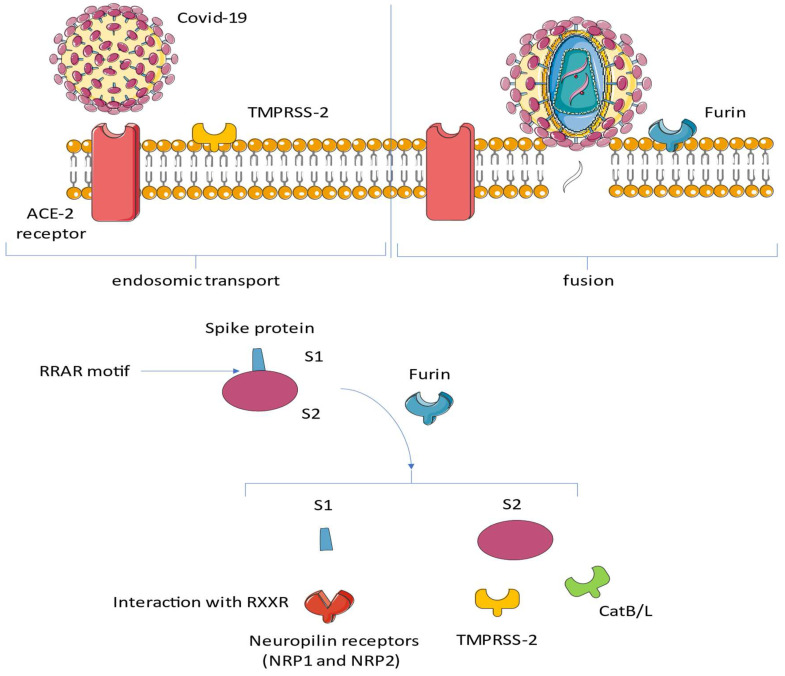
SARS Cov-2 has been shown to possess a marked tropism for cells expressing the ACE2 receptor on their surface. This tropism is mediated by the expression of specific proteases that act synergistically with ACE2. After binding of the spike glycoprotein (S) to the receptor, transmembrane serine protease 2 (TMPRSS2) and TMPRSS13 cleave the full-length spike protein (S0). This reaction promotes the conversion at its S2 site through a complex mechanism mediated by the selective function of the host’s furin. Neuropilin 1 (NRP1) binds the RRAR motif’s carboxyterminal sequence of the spike exposed after furin cleavage. Abbreviations: ACE, angiotensin-converting enzyme; CatB/L, cathepsin B/L; NRP1, neuropilin; S, spike; RAR, RaTG13 virus lacks the furin cleavage motif RRAR↓S; and TMPRS, transmembrane protease, serine.

Accumulating evidence has described the role of a group of SARS-like CoVs (SL-CoV(s)) that have been recognized in horseshoe bats. SL-CoV and SARS-CoV have been noted to share similar genomic arrangements and high-sequence congruities, although a greater derogation is represented by the N-terminus of the spike (S) protein, known to be responsible for receptor binding in CoVs, which constitutes the crucial difference. Lubbe and colleagues [60] provided a comparative analysis of methodologies and findings to describe how structural biology techniques like X-ray crystallography and cryo-electron microscopy have enabled remarkable discoveries into the structure–function relationship of ACE and ACE2. This, in turn, has enabled the development of ACE inhibitors for the treatment of cardiovascular disease and candidate therapies for the treatment of COVID-19. However, despite these advances, the function of ACE homologues in non-human organisms is not yet fully understood. ACE homologues have been discovered in the tissues, body fluids, and venom of species from diverse lineages and are known to have important functions in fertility, envenoming, and insect–host defense mechanisms. Oudit et al. [61] studied the autopsy findings of 20 patients who died during the SARS epidemic in 2003 and found viral RNA genomes in the hearts of seven of them. SARS-CoV can cause serious myocardial inflammatory reactions with damage related to a down-regulation phenomenon at the level of the myocardial angiotensin-converting enzyme 2 (ACE2) system. This action can mediate myocardial dysfunction and unfavorable cardiac outcomes in patients with SARS. Autopsy specimens of myocardial tissue, immunohistochemically stained using a specific macrophage marker (CD68), recorded a significant amount of inflammatory macrophage infiltrate. This cell line was considerably raised in patients who had evidence of SARS-CoV after performing PCR on their hearts, with only a slight increment in those without SARS-CoV. When immunohistochemical staining for T cells was performed, specific cell surface markers (CD3) observed minimal myocardial lymphocyte infiltration with no difference in lymphocyte count between those with and without SARS-CoV in the heart. Although ACE2 is the substantial gateway of viral entry for both SARS-CoV and SARS-CoV-2, different organ tropisms, such as lung and heart, can be expressed and are related to the different clinical presentations and the infectivity related to the two pathogens (Figure 3) [61].

Bat coronavirus RaTG13 is a SARS-like beta coronavirus that infects the horseshoe bat Rhinolophus affinis. It was discovered in 2013 in bat droppings from a mining cave near the town of Tongguan in Mojiang County in Yunnan, China. In February 2020, it was identified as the closest known (at the time) relative of SARS-CoV-2, the virus that causes COVID-19, sharing 96.1% nucleotide similarity.

### 6.2. Immune Response during Myocarditis. Is it Possible to Transfer Knowledge?

**Figure 4:**
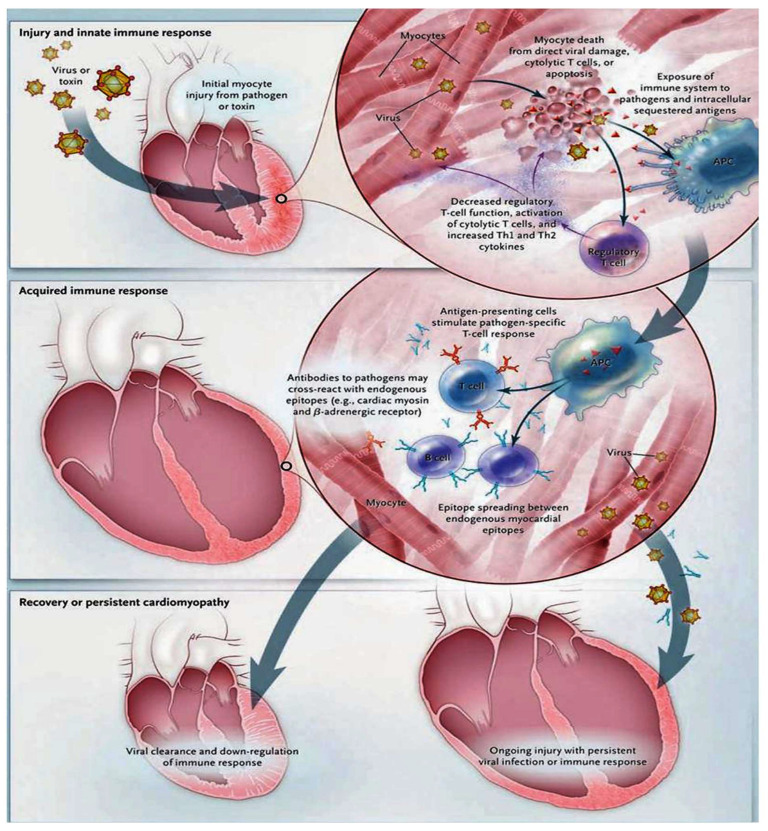
This figure illustrates the immunological mechanisms underlying the pathogenesis of myocarditis. The knowledge we have for understanding the cellular and molecular pathogenesis of post-viral and autoimmune myocarditis is substantially based on animal models. From these models, it emerges that the progression from acute injury to chronic dilated cardiomyopathy can be restricted to a process that includes three phases. In the first stage, acute injury promotes cardiac injury sustained by exposure to intracellular antigens such as cardiac myosin. The activation of the innate immune system activates the subsequent acute inflammatory phase, which develops over the course of several weeks. In this phase, specific immunity mediated by T lymphocytes and antibodies directed against pathogens and similar endogenous cardiac epitopes is triggered, causing a strong inflammatory response. Although most patients succeed, with the available immune defense mechanisms, in eliminating the pathogen, a downregulation of the immune reaction follows with the development of minor sequelae. However, in other patients, virus clearance does not occur, leading to persistent myocyte damage. Heart-specific inflammation persists and tends toward chronicity, which is furthered by the misrecognition of endogenous cardiac antigens as pathogenic entities. Abbreviation: APC, denotes an antigen-presenting cell [62,63,64,65,66,67,68,69,70,71,72,73,74,75,76,77,78,79,80,81,82].

We learned that the innate immune response constitutes the crucial forward front for host defense in the initial phase of infection, as noted in Figure 4.

Not only viruses but also streptococcal M proteins, as well as some host proteins, are capable of promoting an effective innate immune response. This response is regulated by several mechanisms, involving toll-like receptors and pattern-recognition receptors in individuals with infectious tissue lesions [62]. Knowledge in the field of immunology informs us that the development of myocarditis requires MyD88, which appears to be a protein with a substantial role in varied -cell toll-like receptor signaling [63]. For example, a Coxsackievirus B infection induces a specific immunoreaction. Increased regulation of the toll-like receptor 4 on macrophages has been observed, signifying a stimulus towards the maturation of antigen-presenting cells, an increase in the release of proinflammatory cytokines [64], and a decrease in the regulatory function of T cells [65]. It has also been suggested that the increase in production of T helper type 1 (Th1) and T helper type 2 (Th2) cytokine levels, which can be recorded from 6 to 12 h after triggering an innate immune response, was accompanied by the evolution of cardiomyopathy [66]. Therefore, the concerns related to the nature of the innate immune response are crucial in defining the subsequently acquired T and B lymphocyte responses. One point that is not fully elucidated is the autoreactive immune response and whether this promotes viral clearance and regular cardiac function or eventually promotes progression to chronic immune-mediated cardiomyopathy in individual patients. CD4+ T lymphocytes have been reported to play a key role in cardiac tissue damage in experimentally-induced autoimmune myocarditis [67,68]. For example, circulating T cells are normally harmless and exhibit low avidity for self-antigens. However, they may favor the development of immune-mediated heart disease when stimulated with great levels of self-antigens [69]. Immune-stimulated T cells respond with the genesis of both Th1 and Th2 cytokines that have been involved in the pathogenesis of myocarditis following viral infection [70]. A third T helper subset, identified as Th17 cells, characterized by the production of interleukin-17 [71], has been implicated in the pathogenesis of myocarditis [72]. A mouse model was used to demonstrate the crucial role played by both CD4+ and CD8+ T lymphocytes in advocating coxsackievirus B myocarditis [73]. The well-established role of T lymphocytes reported in experimental models of myocarditis has supported the rational use of anti-lymphocyte T therapies in those patients exhibiting severe forms of cardiomyopathy with leading autoimmune features [74]. A subgroup of regulatory T lymphocytes (T reg) plays a role in controlling circulating CD4+ T lymphocytes [75]. Ono and colleagues [76] in an experimental model defined the role of the transcription factor forkhead box p3 (FOXP3) and its interaction with a subset of regulatory T lymphocytes expressing CD4. Possessing a high level of corticosteroid-induced tumor necrosis factor receptor has been shown to affect the progression of autoimmune myocarditis. The roles of CD4+, CD25+, forkhead box P3 (FOXp3), and T lymphocytes was found to be crucial in negatively regulating the inflammatory process induced by coxsackievirus B myocarditis [77]. However, the evaluation of the role of regulatory T lymphocyte subsets in human myocarditis lacks data.

The existence of autoantibodies against a variety of cardiac antigens has been demonstrated in both suspected or histologically confirmed lymphocytic myocarditis and dilated cardiomyopathy [78,79]. The sharing of cardiac myosin epitopes is well known with streptococcal M protein and coxsackievirus B. It has been shown that this antigenic mimicry can evoke the production of autoantibodies through an interaction between intracellular antigen and cross-reactive antibodies [80]. Once the virus has been admitted into the cardiomyocyte, an endogenous source of antigen in chronic myocarditis may be represented by cardiac myosin, which can promote and stimulate chronic inflammation through autoimmune mechanisms. Several studies performed in recent years have reported the phenomenon of cross-reactivity between cardiac myosin and laminin, which functions as an endogenous protein of the human cell surface, to be continuously supportive of chronic myocarditis [81,82]. Li and colleagues [80] noted that antibodies to cardiac myosin cross-react with the β1-adrenergic receptor; additionally, Huber and colleagues noted that these antibodies may promote cardiomyocyte apoptosis [83]. Although antibody autoreactivity commonly occurs in the course of normal immune reactions, discriminating this phenomenon from autoimmune disease, in which anti-cardiac antibodies may cause cardiomyopathy, constitutes a crucial challenge for investigators [82].

### 6.3. Experience from COVID-19 Anatomopathology

In the early stages of the pandemic’s spread, nine cases of myocarditis were described in patients who experienced COVID-19 with positive PCR tests and who received EMBs [84,85,86,87]. As far as the diagnostic criteria were concerned, these were incomplete for the diagnosis of myocarditis, with seven of them being satisfied in two subjects. In all studies, direct evidence of SARS-CoV-2 within cardiomyocytes could not be documented. Furthermore, for most cases, the direct evidence that SARS-CoV-2 infects myocytes by promoting necrosis induced by the direct action of the virus associated with cell death supported by the release of troponin was missing. Sala and colleagues [84] have provided evidence for immune-mediated myocarditis in a patient with COVID-19. The SARS-CoV-2 infection was a confounding factor in the case concerning a 43-year-old woman who presented with a Takotsubo pattern, which was inverted in a setting related to the clinical manifestation of COVID-19. The final diagnosis was explicit virus-negative immune-mediated myocarditis, supported by PCRs of samples obtained after an EMB, which were negative for the presence of the SARS-CoV-2 genome. Escher and colleagues [86] reported the case of a 48-year-old male hospitalized with heart failure in the context of a COVID-19 infection diagnosed with PCR viral molecular testing. The patient underwent an EMB [86], which, upon histological evaluation, met the Dallas criteria for the diagnosis of myocarditis, revealing pronounced myocyte necrosis and a robust inflammatory response of the myocardium with marked infiltration of macrophages and lymphocytes. A PCR test performed on samples of cardiac tissue collected after the EMB noted that the SARS-CoV-2 genome was detectable even if very low levels of gene expression were observed.

Table 1 shows the most relevant evidence obtained from the anatomopathological examination carried out on cardiac tissue samples collected after an EMB or an autopsy in patients who suffered from COVID-19 [25,26,27,84,85,86,87,88,89,90,91,92,93,94,95,96,97,98,99,100,101].

These findings from the examination of the autopsy specimen favored the diagnosis of myocarditis in subjects with COVID-19 in which the virus had infected myocardiocytes. Nevertheless, it is important to emphasize that many of the results were derived from examinations performed on the lung autopsy findings of patients who died from COVID-19. As a matter of fact, few studies have been performed that exclusively select for primarily the expression of COVID-19 in the heart with consequent evidence of viral load. The largest body of evidence was obtained by assessing a series of 25 autopsies conducted at NYC’s Mount Sinai in patients hospitalized for severe forms of COVID-19 [96]. Cardiac atherosclerosis and hypertension were the most frequent pathoanatomical lesions in most of the hearts examined. In 60% of cases, the presence of non-specific tissue alterations with the appearance of patches was revealed and associated with a mild inflammatory response, located primarily in the interstitial compartment of the myocardium, as well as with a lack of myocytic necrosis documented by histological examination.

The pivotal study by Lindner and colleagues [25] first evaluated viral load in the autopsy findings of hearts retrieved from 39 patients who died of a severe episode of COVID-19 [6]. The SARS-CoV-2 genome was found in 24 of the 39 isolated specimens, and in these 61.5%, a precise quantification of the viral load was provided. Despite having robust histological material, the investigators were unable to confirm the diagnosis of myocarditis in compliance with the completeness dictated by the Dallas criteria. Basso and colleagues [100] also worked on a considerably large number of cardiac autopsies obtained from different centers. In this multicenter report on 21 specimens examined, myocarditis was found in only three cases (14.2%) and was described as an inflammatory infiltrate in which myocyte damage not attributable to another aetiology coexisted with a characteristic of multifocality [100]. Immunohistochemistry in the three cases revealed an inflammatory infiltrate consisting of lymphocytes and macrophages. The presence of CD3-positive lymphocytes was detectable in 100% of cases; two findings supported the presence of CD4; and in one specimen retrieved, the predominance of CD8 was reported. The findings in this research are supported by a decidedly higher myocarditis rate than evidenced in other studies, and this fact was due at least in part to the assessment based on the reports provided by four centers, minimizing the concerns related to selection bias.

Much of our knowledge in the field of autopsy-based diagnosis of heart myocarditis is derived from a literature review that included 22 reports [102]. In this analysis, a substantially larger number (*n* = 277) of heart autopsies were evaluated. Myocarditis was observed on initial examination in 20 hearts (7.2%). This first part of the analysis was judged insufficient, and the authors suggested that most of the cases explored were probably not functionally significant. Proceeding to a more careful examination, the authors were able to ascertain that the prevalence of myocarditis was decidedly lower, not exceeding 2% [102]. The anatomopathological evidence was referred to in the cardiac biopsies and autopsy findings of subjects suffering from a severe form of COVID-19. Specific and well-adopted criteria for the diagnosis of myocarditis have been discussed in the myocarditis descriptive articles reported in Table 1. A total of 201 collected hearts, which included EMB samples, were examined and discussed in their article reporting irrefutable evidence for the diagnosis of myocarditis. The evidence provided by the investigators relates to myocarditis of unclear extent and nature, and only nine such cases reached the congruence needed to establish definite evidence of myocarditis. These results suggest that most of the cases described were not supported by substantial evidence about the presence of the virus in the heart. Therefore, in most of the evaluations performed, it was not evident that the myocytes were directly infected by the virus.

A key point that cannot be deferred lies in the crucial caveat regarding a correct evaluation of these studies, which also includes the clinical diagnosis of SARS-CoV-2. In all these reports, the possibility of having false negative results must be taken into consideration, as can emerge in the case of tissue sample analysis. Woloshin and colleagues [102] discussed how sampling errors involving a nasopharyngeal swab can be inaccurate in two ways. A false positive result erroneously labels a person infected, with consequences including unnecessary quarantine and contact tracing. False negative results are more consequential because infected persons—who might be asymptomatic—may not be isolated and can infect others. In particular, false negatives ranged from 2 to 29% due to possible degradation of viral RNA during the fixation process that precedes sample analysis. However, the certainty of the evidence was considered very low because of the heterogeneity of sensitivity estimates among the studies, the lack of blinding to index-test results in establishing diagnoses, and the failure to report key RT-PCR characteristics. Taken as a whole, the evidence, while limited, raises concern about frequent false negative RT-PCR results [103].

This concern emerges clearly in the report by Wenzel et al. [87], in which two cases of COVID-19 that were negative for the nasopharyngeal molecular swab are described. Subsequently, with the use of PCR, the viral genome was extracted and sequenced from the EMB samples. This result highlights the importance that must be given to the reliability of RNA tests of the SARS-CoV-2 genome, necessary for the complete examination of the diagnostic pathological reports. Despite the possibility of obtaining false-negative PCR results, while tissue sampling has been demonstrated, the likely overall reduced expression of the ACE2 receptor in myocardial cells should also be taken into consideration. This factor may promote reduced tropism of SARS CoV-2 for the heart with a deficit in the detection of the viral genome [104]. Finally, Takotsubo syndrome plays a crucial role in the differential diagnosis of SARS-CoV-2 viral myocarditis, which can manifest as a myocardial lesion associated with electrocardiographic abnormalities and increased plasma troponin levels, as observed in normal coronary arteries. On echocardiographic examinations, a regional wall motion abnormality can be found, while the autopsy specimens examined demonstrated myocytolysis and interstitial inflammatory infiltration of the myocardium by lymphocytes and macrophages. However, a diagnosis of exclusion of myocarditis is necessary to safely reach that of Takotsubo syndrome, also because troponin levels and CMR signs of edema can be detected in both clinical conditions, which is a cross-imitation [105,106].

## 7. Clinical Experience

Zhang and colleagues evaluated [56] a large panel of 384 autopsies, of which 239 were cardiac findings, 51 were non-cardiac findings, and the remaining 94 specimens were collected from subjects who died of natural causes. From the totality of the samples, 18% of the inflammatory infiltrates were localized in the heart, and 9% of these constituted multifocal inflammatory lesions. Crucial data was offered by the accidental infiltrates, which revealed a high-frequency rate of 31% in the autopsy findings attributable to natural, non-cardiac deaths and therefore confounding factors of unapparent cardiac involvement. These findings are suggestive when compared to the 20% reported in cardiac inflammatory infiltrates observed in drug-related deaths, the 16% reported in cardiac deaths, and the same 16% reported in autopsy specimens of trauma-related deaths [21]. Anderson et al. [55] noted that the same diagnostic difficulty seen for viral myocarditis in patients with COVID-19 was described in the analysis of autopsied heart samples from subjects succumbing to AIDS-related viral infections. In 31% of these cases, the presence of an attenuated and focal inflammatory infiltrate was observed, which was not sufficient to establish the diagnosis of cardiac viral myocarditis as per the Dallas criteria. The authors observed that mild focal inflammatory infiltrates characterized by mononuclear cells developed suddenly in 10 of 24 (42%) cardiac autopsy findings of subjects who died of traumatic deaths. In these cardiac foci, it was impossible to demonstrate necrosis of the myocytes as the primary lesion to diagnose myocarditis [55].

A considerable contribution to the understanding of viral myocarditis was derived from the Italian experience gained in the province of Bergamo in the Lombardy region, which was one of the European epicenters during the first few months of the SARS-CoV-2 pandemic. From 15 cardiac autopsies of subjects who died at Papa Giovanni Hospital, Bergamo, Italy, and one case from the physician in Baltimore, Maryland, they were evaluated at the CVPath Institute (Gaithersburg, Maryland) for a detailed pathological assessment [29]. Approximately 69% of the autopsy findings were recovered from men, and the mean age of the analyzed cases was 70 years. Antecedent cardiovascular surgery was reported in four cases, while the medical histories of the other four subjects did not highlight relevant pathologies. The severe form of COVID-19 was noted in all cases, as evidenced by the relatively short average duration of hospitalization until death (6 days). The cardiac autopsy was complete with a detailed evaluation, including histological sections retrieved from four walls and at two levels of the heart. None of the autopsy findings examined fully met the necessary criteria to establish a definite diagnosis of myocarditis, albeit in three cases where an acute myocardial infarction was established with certainty. In this context, it was possible to appreciate a hypercoagulative process with thrombi that were localized both in the microvascular bed and in the macrovascular epicardial district. In all these cases, the picture described above was fueled by a conspicuous neutrophilic cellular infiltration. It is important to underline that in 63% of cases, infiltration of inflammatory cells was observed with epicardial involvement, which concerned 10 of the 16 autopsy samples. The infiltration was mainly supported by the discovery of lymphocytes, although in 31% of the cases, the mononuclear inflammatory cell infiltration was noted at myocardial autopsy (five of the 16 examined).

The results of the search for viral RNA have been staggering. One sample from each chamber of the heart for each of the 16 subjects was used to extract total myocardial tissue RNA. Once drawn, the quality and concentration of the RNA samples were calculated. This step involved using a specially designed primer for SARS-CoV-2 (N1 and N2 from the CDC’s EUA assay) so that quantitative reverse transcriptase PCR could be performed. Ramlall and colleagues [107] describe the process by which viral copy numbers were quantified on the standard of the reverse transcriptase PCR results, with the standard check comprising the whole nucleocapsid gene from SARS-CoV-2. RNase P was used as a control. The SARS-CoV-2 RNA revelation was fixed by the amplification at Ct ≤ 40. The virus was detected in only two cardiac autopsy findings by PCR tests, and in both cases, it was found at the atrial level, both in the left and right atria, while the ventricular myocardiocytes were not involved. No inflammatory infiltrate was observed in either atrial site of virus detection. This finding contrasted with evidence of an inflammatory infiltrate observed in most of the 16 cardiac samples and with virus uptake in lung cells.

Kawakami et al. [29] also analyzed the nature of the cells that make up the inflammatory infiltrate in the myocardium of subjects who died from infectious or traumatic causes; the latter constituted the control group, compared to COVID-19 mortalities. The investigators examined, through random selection, the histological sections of the left ventricles of five cases extracted from the register of the deceased, which were compared with five randomly selected cases of subjects who had died of the severe form of COVID-19. Sections of the left ventricular myocardium using antibodies against CD3 (T cell marker) and CD68 (macrophage) were stained. Overall, the examination revealed no difference in the total number of T cells and macrophages between the two groups. However, cell count measurements in the hearts of the COVID-19 deceased were higher than usual for diagnosing myocarditis. The explanation for this finding could be offered by the sparse distribution of cells that were not associated with necrotic myocyte necrosis. The presence of both findings does not provide certainty regarding the criteria for myocarditis. With regards to cell typing, CD3s were significantly more frequent in control autopsy findings than in COVID-19, compared to macrophages, which were more represented in COVID-19 than in the control cases, although it was not possible to assign an exact location of the cells between the interstitium and the intravascular compartment [29].

Lindner [25] examined 39 consecutive autopsy cases, reporting the existence of the viral genome in 24 retrieved autopsy findings of myocardial tissue, while 15 specimens (38.5%) did not reveal SARS-CoV-2. Pneumonia was the cause of death for 89.7% of subjects, and none of the patients described revealed clinically fulminant myocarditis. The presence of the viral genome in myocardial tissue was suggestive of the SARS-CoV-2 spike glycoprotein selective ACE2 receptor expression on the surface of myocardial cells [108], as well as elucidating the crucial collusion of myocardial tissue in infection [109]. After in situ hybridization of myocardial tissue pointed out that the most likely localization of SARS-CoV-2 was not in cardiomyocytes but the virus overrunning interstitial cells or macrophages in myocardial tissue. The existence of CD3+, CD45+, and CD68+ was noted, although the specimens with the viral genome did not disclose an increase in mononuclear cell infiltrates into the myocardium as compared to the autopsy samples without the virus. Of note, in 1/3 of patients with viral loads greater than 1000 copies, which was deemed clinically important, viral replication within myocardial tissue was observed. Moreover, with these levels of viral load, a greater expression of cytokines has been reported, suggesting a substantial role in the modulation of the inflammatory process. Indeed, increased expression of 6 proinflammatory genes related to cytokine production (tumor necrosis growth factor α, interferon γ, chemokine ligand 5, and interleukin-6, -8, and -18) was found in 16 patients compared to 15 patients in whom SARS-CoV-2 was not detected in the heart [25].

The result reported by Lindner and colleagues corroborated the findings of Guzik et al. [110] that related cytokine-induced organ dysfunction to the disease process. What emerges from Lindner’s report is crucial in supporting the fact that patients with SARS-CoV-2 infection associated with viral replication did not have a direct correlation with fulminant myocarditis. In this study, a lack of significant changes in transendothelial migration of inflammatory cells was observed when the myocardial tissue evaluation of autopsy specimens with high viral loads was compared with those that did not have the virus. Conversely, several studies have suggested a correlation between the onset of myocardial inflammation and evidence of clinical myocarditis. In some patients with or without pre-existing cardiovascular comorbidities, myocardial damage characteristic of myocarditis may occur in patients with COVID-19 [110]. Again, after the well-documented case of acute myocarditis following a respiratory infection associated with COVID-19 in a 53-year-old Italian woman, several studies have documented that direct viral infection of the myocardium was a possible causal route of myocardial damage [111]. Ultimately, Lindner and colleagues [25] support the specific condition that viral replication and myocarditis may not be two conjoined processes. Furthermore, their results did not report an increase in inflammatory cells in consecutive cases of COVID-19 without clinical myocarditis. The long-term effects of the presence of SARS-CoV-2 in myocardial tissue appear to play a considerable role. Solid data explaining the pathoanatomical processes related to the presence of viral activity in the myocardium in the absence of clinical symptoms of myocarditis remains elusive. However, it has been observed that the leukocytopenia detectable in patients with COVID-19 could hinder the migration of activated mononuclear cells [11,112]. Low concentrations of macrophages, responsible for the digestion of neutrophil extracellular traps (NETs), could play a crucial role in maintaining a high level of NET release in the myocardial tissue by maintaining a high proinflammatory and procoagulant level [42,43,44,45,46].

Pellegrini and colleagues [113] reported a pathological analysis of 40 hearts from hospitalized patients who died of COVID-19 in Bergamo, Italy. Of a total of 40 hearts assessed, in 14 cases (35%) myocyte necrosis, predominantly at the left ventricle was observed. Subjects with necrosis were more likely to be female, have chronic kidney disease, and have fewer symptoms from onset to admission. A similarity was noted for the incidence of severe coronary artery disease, referred to as >75% cross-sectional narrowing, between the two groups with and without necrosis. In three of the 14 (21.4%) subjects exhibiting myocyte necrosis, acute myocardial infarction was noted and was defined as an area of necrosis ≥1 cm^2^. Instead, in 11 of the 14 subjects (78.6%), focal myocytic necroses with an area ≥0.05 mm^2^ but <1 cm^2^ were found. The presence of heart thrombi was observed in 11 of the 14 (78.6%) subjects with necrosis and two of the 14 (14.2%) cases with evidence of thrombi in the epicardial coronary artery. On the other hand, the presence of microthrombi was found in myocardial capillaries, arterioles, and small muscular arteries in nine out of the 14 cases (64.3%). Cardiac microthrombi from postmortem cases positive for COVID-19 were compared with intramyocardial thromboembolism from COVID-19 cases. A comparison was made between aspirated thrombi obtained during primary percutaneous coronary intervention from both uninfected and COVID-19-infected patients presenting with ST-segment elevation myocardial infarction. The microthrombi examined revealed significantly greater C5b-9 immunostaining of terminal complement and fibrin than in intramyocardial thromboembolism from COVID-19-negative subjects and in those obtained from subjects with aspirated thrombi. No significant differences were found between the constituents of aspirated thrombi from COVID-19-positive and negative patients with ST-segment elevation myocardial infarction.

**Figure 5:**
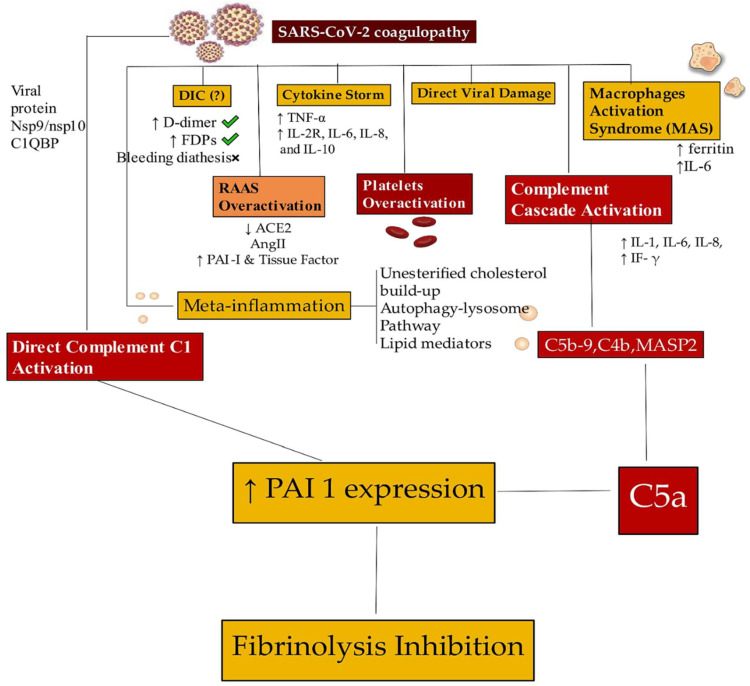
Only a residual proportion of patients with COVID-19 have a diagnosis of viral myocarditis documented by the presence of SARS-CoV-2 in myocardiocytes. The most frequent lesions are induced by the development of micro- and macrothrombi. In patients with COVID-19, micro- and macrothrombotic formations localized both in the microcirculation and in the epicardial vascular system of the heart have been observed. SARS-CoV-2 infections promote dysregulation in the coagulation system, and several mechanisms are implicated. The coagulopathy is supported by the DIC, the cytokine storm process, and the direct action of the virus, which proves the damage and activation of macrophages. Hyperactivation of the RAAS associated with platelet and complement hyperactivation (direct and indirect) leads to inhibition of fibrinolysis. Abbreviations: ACE2, angiotensin-converting enzyme 2; C, complement; DIC, disseminated intravascular coagulation; FDP, fibrinogen-derived peptides; IFN-γ, interferon gamma; IL, interleukin; MAS, macrophage activation syndrome; PAI, platelet activator inhibitor; TNF, tumor necrosis factor; RAAS, renin-angiotensin-aldosterone system. The arrows explain the increase or decrease of the related component. ↑, increases; ↓, decreases. When to do the endomyocardial biopsy [42,43,44,45,46,47,48].

These findings all confirm the results proven from autopsy specimens of subjects needing the diagnosis of myocarditis. The most common pathological cause of myocyte necrosis was the production of microthrombi. Differences were found at the level of microthrombi that were different in composition from intramyocardial thromboembolic formation retrieved from COVID-19-negative subjects and coronary thrombi recovered from COVID-19-positive and -negative patients with ST-segment elevation myocardial infarction (Figure 5).

An endomyocardial biopsy is not free from potential intrinsic risks, including cardiac perforation, which involves those regions of the heart made fragile by inflammation or treatment with steroid drugs, pericardial tamponade, and bleeding. The use of flexible bioptomes characterized by the incorporation of smaller jaws has had a considerable impact on reducing the risk of complications. Cooper and colleagues [17] reported a complication rate of 1.2%, and among these, the most frequent is the perforation of the heart, which reaches a percentage of 0.42%, which can be associated with the death of the recipient in 0.03%.

**Figure 6:**
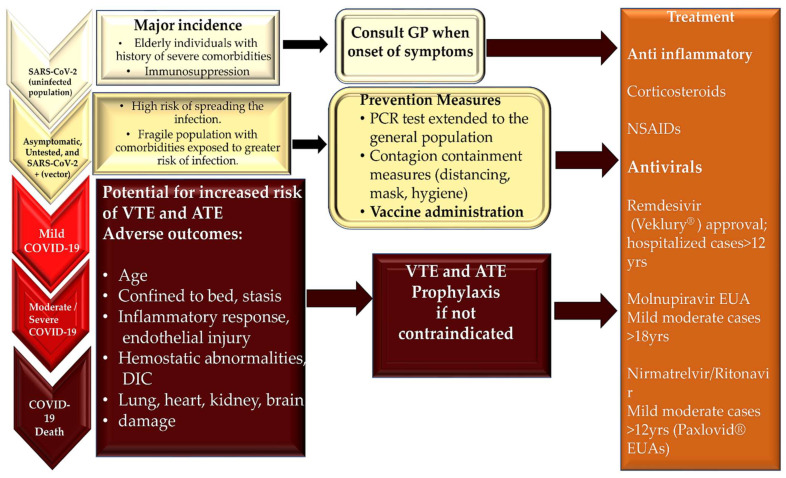
Patients with COVID-19 have different clinical evolutions. Their treatment options depend on the diagnosis of the complications that occur. Clinical variability in the manifestation of COVID-19 has been observed in the population developing SARS-CoV-2 infections. Myocarditis occurs rarely compared to thrombosis; however, both are driven by inflammation. The inflammatory response increases with age and bed rest, which is more frequent in severe COVID-19, and may contribute to thrombosis and adverse events resulting from multiorgan involvement. The FDA’s timeline for the approval of antivirals and the EUA’s process timeline. Veklury^®^ EUA’s process was formalized in January 2020. Its final approval occurred in October 2020. Molnupinavir and Paxlovid^®^ EUA’s process followed in December 2021. Abbreviations: ATE, arterial thromboembolism; COVID-19, coronavirus disease 2019; DIC, disseminated intravascular coagulation; EUA, Emergency Use Authorization; FDA, Food and Drug Administration; NSAIDs, non-steroidal anti-inflammatory drugs; SARS-CoV-2, severe acute respiratory syndrome coronavirus 2; and VTE, venous thromboembolism.

Taking into account all these determinants, the indication for an EMB approach should consider the probability and risk of sampling error to anticipate the expected yield. EMB is closely linked to the existence of effective therapy. Considering all these aspects, the procedure should be performed only when the benefits, including that of an adequate antiviral treatment to be administered immediately, far outweigh the risks associated with the approach. The diagnosis of myocarditis is rare in patients with COVID-19, as observed by the preponderance of autopsy examinations performed and EMB results obtained in suspected cases of SARS-CoV-2 myocarditis. The first concern related to the use of an EMB is closely related to the procedure that involves the part of the heart that is reached by the bioptome to obtain the sampling, which is generally limited to the region of the heart corresponding to the right ventricular septum. This aspect influences the result by restricting the effective diagnostic yield and making the diagnostic result improbable. Numerous publications have noted this in suspected cases of myocarditis in the context of a clinical picture of COVID-19. [25,26,27,87,88,89,90,94,95,96,97] (Table 1). The second concern is that histological diagnosis is related to the different inflammation patterns that set typical criteria for myocarditis, which may be different in patients with COVID-19 because these subjects tend to have lymphopenia [20,21,25,27,29,114]. Indeed, there is no data to indicate the extent of lymphocytic infiltration that should be found in patients with suspected typical COVID-19 myocarditis. The third concern relates to the therapeutic implications that are not supported by robust evidence, as there is insufficient data to support the efficacy of specific myocarditis therapies in patients exhibiting COVID-19. To date, substantial evidence advocating the routine approach with an EMB is scarce, so the procedure is not recommended in patients with a suspicion of myocarditis, and it is believed that it should be reserved for patients with a more severe clinical picture. This is the case of subjects who develop fulminant heart failure with compromised hemodynamics in the context of a SARS-CoV-2 infection and a severe form of COVID-19 documented by PCR tests in the absence of coronary artery disease [89] (Figure 6).

Infection caused by SARS-CoV-2 potentially carries a marked morbidity that can lead to high mortality, especially in patients with an associated cardiac injury. However, the mechanism by which the virus advocates cardiac damage is not yet fully understood. The data emerging on the cause-and-effect relationship between viral infection and COVID-19-induced myocarditis suggest that myocarditis has a substantial role in promoting the mechanism responsible for the damage in a small number of cases that reach a certain diagnosis. [29]. The procedures adopted to arrive at the diagnosis of myocarditis have revealed that the diagnosis of myocarditis is acquired in only 4.5% of those examined, with highly selected cases subject to autopsies or an EMB (central illustration). The use of an EMB is recommended by current guidelines and the position papers of professional societies, predominantly on the basis of large observational studies that have reported a benefit with regard to the diagnosis of myocarditis after an EMB [17,19]. If the postponement bias of autopsy studies is considered, the number of COVID-19 cases complicated by myocarditis is still lower than reported. This occurrence depends both on the fact that a large proportion of myocarditis is not complicated by death and on the lack of substantial evidence of the existence of myocardial damage. It has also been observed that an inflammatory substrate of the myocardium characterized by macrophage and T cell infiltration can be found in autopsy findings of deaths from non-infectious causes and autopsy specimens retrieved from COVID-19 deceased subjects, although the extent of infiltration is different from case to case [20,21,25,26,27]. Again, these results are not definitive for clinically relevant myocarditis. 

It is important to point out that in the hearts of subjects with SARS-CoV-2 infections, a non-specific inflammatory infiltrate was found in which macrophages were more abundant than T cells. These results conflicted with the data found in the autopsy findings of control hearts, where a greater presence of lymphocytes was recorded compared to macrophages. (Graphical Abstracts). This evidence may be consistent with the known lymphopenic effects of the virus, although it needs further exploration [20,21,25,26,27,96,99,100,101].

## 8. Treatment

The therapy for acute myocarditis that develops in patients with COVID-19 is aimed at treating heart failure that results from dilated cardiomyopathy and arrhythmias that can arise as a consequence of the established cardiac inflammatory substrate and heart failure development. In patients suffering from myocarditis with acute dilated cardiomyopathy, the recommended treatment is directed according to current guidelines from the ACC/AHA, the European Society of Cardiology (ESC), and the Heart Failure Society of America [115,116]. Similarly, the medical treatment of symptomatic SARS-CoV-2 (COVID-19) infection in the early stages of the pandemic was mainly aimed at containing the inflammatory response of the cytokine storm type.

### 8.1. Heart Failure and Arrhythmias Treatment

The crucial role of therapy for acute myocarditis is to support left-sided ventricular dysfunction. Most patients are treated using the standard treatment regimen for heart failure, which includes the administration of beta-blockers such as metoprolol and carvedilol, angiotensin receptor blockers or angiotensin-converting enzyme inhibitors, and if required, diuretics. The concern is related to clinically worsening patients despite optimal medical management. Several studies have demonstrated the safety and effectiveness of the role offered by mechanical circulatory support, such as ventricular assist devices or extracorporeal membrane oxygenation, as a bridge to recovery or possible transplantation [117,118,119]. Although the overall survival rate after heart transplantation for myocarditis is similar to that for other causes of heart failure [120], there is no data reporting survival after transplantation in subjects who have experienced the severe form of COVID-19 [121].

The establishment of acute myocarditis may involve supportive therapy for the control of arrhythmias, which usually occur in the acute phase of the disease and last for several weeks. The guidelines from the ACC/AHA and the ESC recommend the administration of conventional drugs for arrhythmias that occur in patients with myocarditis [122,123,124]. If symptomatic bradycardia or complete heart block occurs, the implantation of temporary pacemakers may be necessary. The manifestation of symptomatic or sustained ventricular arrhythmias may require the administration of amiodarone, and in some cases, implantable cardiac defibrillators may be necessary.

### 8.2. Antivirals and Immunomodulator Treatment

Even in the pre-COVID-19 era, the therapeutic effects of antivirals and immunomodulators have raised many doubts. It had been suggested in experimental models and uncontrolled case series that intravenous immunoglobulin (IVIG) administration might confer a therapeutic benefit in myocarditis. However, in the Intervention in Myocarditis and Acute Cardiomyopathy study, it was reported that patients who experienced acute dilated cardiomyopathy and received IVIG administration did not benefit from drug treatment compared with those who received a placebo [125]. Given the evidence reported in cases of acute myocarditis in adults, the routine use of IVIG is not recommended. IVIG has not been rigorously evaluated as a treatment for chronic viral inflammatory processes associated with chronic dilated cardiomyopathy and viral inflammation or persistence, regardless of the presence of SARS-CoV-2. On the other hand, IVIG administration was effective in medical treatment for acute pediatric myocarditis [47,126,127], but not noted in pediatric-onset COVID-19.

The results from randomized controlled trials and ESC Guidelines for the diagnosis and treatment of acute and chronic heart failure have demonstrated that immunosuppression for acute myocarditis has proven effective and could fill the therapeutic gap regarding the medical treatment of myocarditis in COVID-19 [116,128]. Evidence has suggested that virus-negative inflammatory cardiomyopathy benefits from immunosuppressive therapy even after long-term follow-up. Recurrence appears to respond to a new TIMIC protocol application [129].

The advent of the pandemic was a sudden phenomenon that found healthcare systems around the world unable to completely handle it, both in terms of the organization of hospital structures and effective therapeutic contributions to deal with the unknown disease. The lack of a large body of scientific literature to support the medical treatment of myocarditis is because acute myocarditis involves few patients, has a highly variable clinical prognosis, and is associated with substantial improvement in left ventricular function with conventional care. The initiation of treatment must therefore take into consideration the following requirements: (1) Clarifying myocarditis must be diagnosed with certainty before starting any therapy. (2) When the definitive histological diagnosis is reached, the etiological treatment must be guided by the indications of the 2013 ESC consensus. In this context, CMR is the reference standard for obtaining the diagnosis of acute myocarditis injury, and it has had early use in patients with COVID-19 after SARS-CoV-2 infection [17,130].

In patients with symptomatic COVID-19, the major proposed treatments consisted of remdesivir and favipiravir as inhibitors of viral RNA polymerases and, subsequently, of viral RNA production, corticosteroids such as dexamethasone, JAK-STAT pathway modulators, and other systemic therapeutic agents. The administration of hydroxychloroquine (an antimalarial agent) alone or in combination with azithromycin is not recommended against COVID-19 [131]. The enthusiasm for the use of hydroxychloroquine caused rapid FDA approval in March 2020 to treat hospitalized patients weighing at least 50 kg. However, this was soon tempered by the results provided by the RECOVERY trial. In this randomized clinical trial (RCT), designed to compare hydroxychloroquine to standard care for 4716 hospitalized patients with COVID-19 disease, the administration of hydroxychloroquine failed to prove any benefit in terms of 28-day mortality [132]. Although other possible non-antiviral agents recommended against COVID-19 have been used at different times of the pandemic, the response has been mixed. These non-antiviral agents include corticosteroids, interleukin-6 (IL-6) receptor antagonists (tocilizumab), and monoclonal antibodies targeting the Janus kinase signal transducer and activator of transcription pathway (JAK-STAT), such as tofacitinib and baricitinib.

In several studies [133,134,135,136,137,138,139,140], the use of interleukin-6 (IL-6) inhibitors, tocilizumab in particular, seemed to decrease clinical deterioration in terms of death, and the need for mechanical ventilation, ECMO, or intensive care unit (ICU) admission. However, an RCT performed to assess the outcome of 130 patients receiving either tocilizumab in intravenous administration or standard supportive care failed to prove a substantial clinical benefit in terms of death after 28 days.

Tofacitinib is an oral inhibitor of the Janus kinase 1 (JAK1) and the Janus kinase 3 (JAK3) enzymes, used in association with methotrexate for the management of active rheumatoid arthritis [141]. Together with other agents such as baricitinib, it interferes with the JAK-STAT signaling pathway to reduce immunopathological reactions by restricting growth factor receptor stimulation [142]. Baricitinib administration has been associated with an increased risk of thromboembolism when compared with a placebo in safety studies for rheumatological diseases and COVID-19 [141,142,143]. Of note is that there is ongoing interest in the use of tofacitinib, which is currently being suggested for patients with severe COVID-19 on supplemental or high-flow oxygen therapy [144].

Evidence for corticosteroid application and efficacy in COVID-19 is generally represented by RCTs. Moderate-level evidence has demonstrated overall mortality reduction in hospitalized patients being prescribed dexamethasone, but other endpoints, such as ventilator-free days, a new need for invasive ventilation, and quality of life parameters were statistically limited by low patient numbers to draw any meaningful conclusions [145,146,147,148,149,150]. In mild diseases, case numbers were insufficient to derive conclusions. Dequin et al. [147] evaluated the effects of hydrocortisone administration on 21-day mortality in critically ill patients. The study was terminated with a low number of patients, like other similar studies (149 enrolled), and low-dose hydrocortisone was demonstrated as non-superior to a placebo, with the primary outcomes being mortality, mechanical ventilation use, and high-flow oxygen therapy. Secondary ones, comparable to the other RCTs, were the need for tracheal intubation, the incidence of pronation, extracorporeal membrane oxygenation, inhaled nitric oxide, the Pao2:Fio2 ratio, and nosocomial superimposed infections [147].

**Figure 7:**
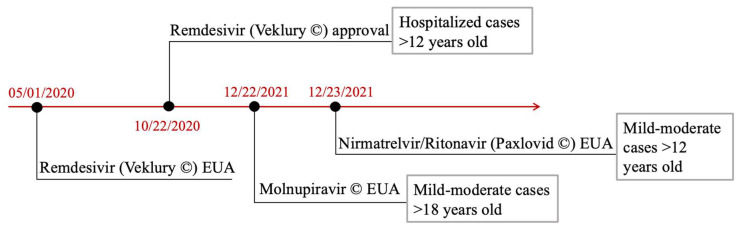
The lack of an established antiviral therapy has tempered the use of an EMB. The figure shows the most commonly used antivirals in patients with COVID-19, with or without the development of viral myocarditis, as well as the different times that led to their approval. The FDA’s and EUA’s process timeline for antiviral approval. Remdesivir (Veklury^®^) EUA’s process was definitively formalized in January 2020, and its definitive approval occurred in October 2020. Molnupinavir and Paxlovid^®^ EUA’s process followed in December 2021. In addition, gray-outlined boxes demonstrate the presented indications on the side of each authorized therapeutic agent. Abbreviations: FDA: Food and Drug Administration; EUA: Emergency Use Authorization [131,132,133,134,135,136,137,138,139,140,141,142,143,144,145,146,147,148,149,150,151,152,153,154].

After Emergency Use Authorizations (EUAs), according to the current clinical evidence, the Food and Drug Administration (FDA) and the European Medicines Agency (EMA)-approved antivirals for COVID-19 treatment are Remdesivir [151], Molnupinavir [152,153], Paxlovid [154], and a combination of nirmatrelvir and ritonavir (Figure 7).

## 9. Conclusions

Infections caused by SARS-CoV-2 potentially carry a marked morbidity that can lead to high mortality, especially in patients with an associated cardiac injury. However, the mechanism by which the virus causes cardiac damage is not yet fully understood. The data emerging on the cause-and-effect relationship between viral infections and COVID-19-induced myocarditis suggest that myocarditis has a substantial role in promoting the mechanism responsible for the damage in a small number of cases that reach a certain diagnosis [29]. The procedures adopted to arrive at the diagnosis of myocarditis have revealed that the diagnosis of myocarditis is acquired in only 4.5% of those examined, and in highly selected cases, they are subject to autopsies or EMBs (central illustration). The use of an EMB is recommended by current guidelines of professional societies, predominantly on the basis of large observational studies that have reported a benefit with regard to the diagnosis of myocarditis after an EMB [17,19]. If the postponement bias of autopsy studies is considered, the number of COVID-19 cases complicated by myocarditis is still lower than reported. This occurrence depends both on the fact that a large proportion of myocarditis is not complicated by death and on the lack of substantial evidence of the existence of myocardial damage. It has also been observed that an inflammatory substrate of the myocardium characterized by macrophage and T cell infiltration can be found in autopsy findings of deaths from non-infectious causes and autopsy specimens retrieved from COVID-19 deceased subjects, although the extent of infiltration is different from case to case [20,21,25,26,27]. Again, these results are not definitive for clinically relevant myocarditis.

It is important to highlight that in the hearts of subjects with SARS-CoV-2 infections, a non-specific inflammatory infiltrate was found in which macrophages were more abundant than T cells. This result was opposite to the data found in the autopsy findings of control hearts, where a greater presence of lymphocytes was recorded compared to macrophages. (Graphical Abstracts). This finding may be consistent with the known lymphopenic effects of the virus, although it needs further exploration [20,21,25,26,27,96,99,100,101].

The conclusions emerging from this review suggest that COVID-19-induced myocarditis is a rare phenomenon compared to the more common finding of microthrombi and macrothrombi in the vascular beds of the heart. Compared with current controls, patients with COVID-19 and elevated cardiac troponin levels revealed more ventricular impairment and myocardial scarring in early convalescence. However, the proportion with myocarditis was observed as low, and scar pathogenesis was substantially different, including a newly described pattern of microinfarction [155]. In this context, the use of CMR to drive diagnosis is consistent due to its high sensitivity and specificity [130,155,156]. Furthermore, the therapeutic implications of failure to identify viral myocarditis in subjects with COVID-19 remain uncertain. The literature highlights that EMBs have not been routinely used because there are no therapeutic certainties from the administration of available antiviral drugs. Therefore, an invasive approach should be reserved for patients manifesting more severe clinical conditions with the recent onset of rapidly worsening heart failure and with hemodynamic compromise in the context of a PCR-documented SARS-CoV-2 infection, which is responsible for suspected fulminant myocarditis in the course of COVID-19.

## Figures and Tables

**Table 1:** Around 202 cases were studied with an autopsy, an endomyocardial biopsy, and CMRI. Myocarditis was diagnosed in 10 cases [25,26,27,84,85,86,87,88,89,90,91,92,93,94,95,96,97,98,99,100,101].

First Author/Year Ref	Type of Study	Total Number of Patients	Autopsy (n) or EMB	Sex	Mean Age, Yrs	H/O Underlying HD, Type	** Findings
Lindner et al. 2020 [25] JAMA Cardiol	Prospective	39	AUT	16 M 25 F	85 (78–89)	17 HTN, 32 CAD, and 7 DM	Myocarditis: 0 Virus Detected: 24 ^†^ Impaired Cardiac Function: NA Type and Cells: NA
Fox et al. 2020 [26] Circulation Fox et al. 2020 [99] Lancet Respir Med	OS	22	AUT	NA	69 (44–79)	18 HTN, 1 CAD, 11 DM, 4 CKD, 9 obesities	Myocarditis: 0 Virus Detected: NA Impaired Cardiac Function: 2/22 HF Type and Cells: Scattered single myocyte necrosis without significant lymphocyte infiltration
Varga et al. 2020 [27] Lancet	OS	3	AUT	2 M 1 F	66 (58–71)	HTN, 1 CAD, 1 DM, and 1 obesity	Myocarditis: 0 Virus Detected: NA Impaired Cardiac Function: 1/3 EF low Type and Cells: 0, Endothelialitis
Sala et al. 2020 [84] Eur Heart J	Prospective	1	EMB	F	43	0	Myocarditis: 1 Virus Detected: No Impaired Cardiac Function: EF 43% Type and Cells: T cell + necrosis (limited)
Tavazzi et al. 2020 [85] Eur J Heart Fail	Prospective	1	EMB	M	69	0	Myocarditis: 0 Virus Detected: Yes Impaired Cardiac Function: EF 34% Type and Cells: Low-grade inflammation, no necrosis
Escher et al. 2020 [86] ESC Heart Fail	Prospective	5	EMB	4 M 1 F	49 (36–62)	NA	Myocarditis: 1 Virus Detected: 5 Impaired Cardiac Function: 4/5 Impaired Type and Cells: Active lymphocytic myocarditis
Wenzel et al. 2020 [87] Cardiovasc Res	Prospective	2	EMB	2 M	39, 36	1 HTN, 1 HF, and 1 CAD	Myocarditis: 0 Virus Detected: 2 Impaired Cardiac Function: EF 60% EF 30% Type and Cells: Lymphocyte infiltration, no necrosis
Pesaresi et al. 2021 [88] Eur Rev Med Pharmacol Sci	OS	1	AUT	F	84	NA	Myocarditis: 0 Virus Detected: Yes (TEM) Impaired Cardiac Function: NA Type and Cells: Virus in myocytes by TEM, no inflammatory cells
Xu et al. 2020 [89] Lancet Respir Med	OS	1	AUT	M	50	0	Myocarditis: 0 Virus Detected: No Impaired Cardiac Function: NA Type and Cells: Interstitial inflammatory cells
Barton et al. 2020 [90] Am J Clin Pathol	OS	2	AUT	2 M	77, 42	1 HTN	Myocarditis: 0 Virus Detected: NA Impaired Cardiac Function: NA Type and Cells: 0
Bradley et al. 2020 [91] The Lancet	OS	14	AUT	6 M 8 F	71 (42–84)	9 HTN, 3 CAD, 4 HF, 8 CKD, 5 DM, and 5 obesities	Myocarditis: 1 Virus Detected: + ℷ Impaired Cardiac: Function NA Type and Cells: Lymphocyte infiltration with necrosis
Buja et al. 2020 [92] Cardiovasc Pathol	OS	23	AUT	12 M 7 F 4 NA	NA (34–76)	10 HTN, 5 DM, and 9 obesity	Myocarditis: 1 Virus Detected: NA Impaired Cardiac Function: NA Type and Cells: Lymphocytic myocarditis
Wichmann et al. 2020 [93] Ann Intern Med	OS	12	AUT	9 M 3 F	73 (52–87)	2 HTN, 6 CAD 2 CKD, 1 PAD, 3 DM, and 3 obesities	Myocarditis: 1 Virus Detected: NA Γ Impaired Cardiac Function: NA Type and Cells: Lymphocytic myocarditis
Lax et al. 2020 [94] Ann Intern Med	OS	11	AUT	8 M 3 F	80 (66–91)	9 HTN, 5 DM, 3 CAD, and 2 Malig	Myocarditis: 0 Virus Detected: NA Impaired Cardiac Function: NA Type and Cells: Focal lymphocytic infiltrate
Tian et al. 2020 [95] Mod Pathol	OS	4	AUT	3 M 1 F	73 (59–81)	1 DM and HTN	Myocarditis: 0 Virus Detected: NA Impaired Cardiac Function: NA Type and Cells: 0
Bryce et al. 2021 [96] Mod Pathol	OS	25	AUT	NA	69 (34–94)	HTN 63%, CAD 31%, and DM 40%	Myocarditis: 0 Virus Detected: NA Impaired Cardiac Function: NA Type and Cells: 2 cases of interstitial chronic inflammation
Beigmohammadi MT et al. 2021 [97] Int J Surg Pathol	OS	7	AUT	5 M 2 F	68 (46–84)	4 HTN, 1 IC 1 DM, and 1 VD	Myocarditis: 0 Virus Detected: Non in 4 cases NA Impaired Cardiac Function: NA Type and Cells: Inflammation and necrosis but no myocarditis
Rapkiewicz et al. 2020 [98] EClinicalMedicine	OS	7	AUT	3 M 4 F	57 (44–65)	6 HTN, 5 DM, and 5 obesity	Myocarditis: 1 Virus Detected: Non in 4 cases (EM) Impaired Cardiac Function: NA Type and Cells: 1 case of focal lymphocytic infiltration with myocardial necrosis
Basso et al. 2020 [100] Eur Heart J	OS	21	AUT	15 M 6 F	69 (44–86)	16 HTN, 7 DM, 3 CAD, and 2 CKD	Myocarditis: 3 Virus Detected: NA Impaired Cardiac Function: 2/21 died due to Cardiogenic shock or cardiac arrest * Type and Cells: Multifocal lymphocyte infiltration with myocardial necrosis
Inciardi et al. 2020 [101] JAMA Cardiol	OS	1	NA	F	53	No history CVD No DM	Myocarditis: 1 Virus Detected: NA Impaired Cardiac Function: EF 35% Type and Cells: NA

** The reported cases of myocarditis have a definite diagnosis according to the authors. Borderline cases of myocarditis were not included in the diagnostic cases of myocarditis. ℷ Very low level of virus was detected by PCR (likely contamination by circulating virus rather than direct infection). Γ 5 patients disclosed the detection of virus in other tissues, although it was not clearly stated in which tissues the virus was detected. * 12 patients showed new ECG abnormalities including atrial fibrillation, premature ventricular beats, bundle branch block, and ST-segment abnormalities. Only 5 cases were evaluated by cardiac ultrasound without any evidence of impaired cardiac function. K The same case series was reported in other papers (PMIDs 32689809 and 32473124). ^†^ Viral load was less than 1000 copies in 8 patients and was above 1000 copies in 16 patients. Abbreviations: Aut, autopsy; CAD, coronary artery disease; CHF, chronic heart failure; CKD, chronic kidney disease; COVID-19, coronavirus disease-2019; DCM, dilated cardiomyopathy; DM, diabetes mellitus; EF, ejection fraction; EM, electron microscopy; EMB, endomyocardial biopsy; HCM, hypertrophic cardiomyopathy; HD, heart disease; HF, heart failure; H/O, history of; HTN, hypertension; IC, immunocompromised; Malig, malignancy; NA, no available information; PAD, peripheral artery disease; TEM = transmission electron microscopy; and VD, valve disease.

## Data Availability

Not applicable.
